# How Does the Waterlogging Regime Affect Crop Yield? A Global Meta-Analysis

**DOI:** 10.3389/fpls.2021.634898

**Published:** 2021-02-19

**Authors:** Li-xin Tian, Yu-chuan Zhang, Peng-liang Chen, Fei-fei Zhang, Jing Li, Feng Yan, Yang Dong, Bai-li Feng

**Affiliations:** ^1^State Key Laboratory of Crop Stress Biology in Arid Areas/Shaanxi Research Station of Crop Gene Resources and Germplasm Enhancement, Ministry of Agriculture, College of Agronomy, Northwest A & F University, Yangling, China; ^2^College of Agronomy, Northeast Agricultural University, Harbin, China; ^3^Qiqihar Branch, Heilongjiang Academy of Agricultural Sciences, Qiqihar, China

**Keywords:** crop type, growth stage, waterlogging duration, grain yield, meta-analysis

## Abstract

Waterlogging, an abiotic stress, severely restricts crop yield in various parts of the world. Thus, we conducted a meta-analysis of 2,419 comparisons from 115 studies to comprehensively evaluate the overall change in crop yield induced by waterlogging in the global region. The results suggested that waterlogging obviously decreased crop yield by 32.9% on average, compared with no waterlogging, which was a result of a reduced 1,000-grain weight (13.67%), biomass (28.89%), plant height (10.68%), net photosynthetic rate (*P*_*n*_, 39.04%), and leaf area index (LAI, 22.89%). The overall effect of a waterlogging regime on crop yield is related to the crop type; the crop yield reduction varied between wheat (25.53%) and cotton (59.95%), with an overall average value of 36.81% under field conditions. In addition, we also found that compared with no waterlogging, waterlogging in the reproductive growth stage (41.90%) caused a greater yield reduction than in the vegetative growth stage (34.75%). Furthermore, decreases in crop yield were observed with an extension in the waterlogging duration; the greatest decreases in crop yield occurred at 15 < D ≤ 28 (53.19 and 55.96%) under field and potted conditions, respectively. Overall, the results of this meta-analysis showed that waterlogging can decrease crop yield and was mainly affected by crop type, growth stage, and experimental duration.

## Introduction

Waterlogging is an identifiable phenomenon where free water overlays the soil surface of cropland (Striker, 2012). It is estimated that 12% of the world’s arable land could be waterlogged frequently, leading to approximately 20% crop yield reduction (Setter and Waters, 2003). In the near future, soil waterlogging is predicted to increase in frequency and magnitude due to global climate change, especially in irrigated regions and under more intense and unpredictable rainfall, such as in the Yangtze Watershed, the plains of Huang-Huai-Hai, Sanjiang, and Songnen in China (Shi and Zhou, 2006; He, 2014), as well as irrigated areas of the United States, India, Pakistan, Argentina, and Europe (Pang et al., 2004; Feyen et al., 2012; Ploschuk et al., 2018). At the same time, soil containing a large amount of clay or soil that is highly compacted due to repeated use of agricultural machinery can have poor drainage, which can also lead to an increased occurrence of waterlogging events (Najeeb et al., 2015; Ploschuk et al., 2018). Therefore, waterlogging is an increasingly important adverse stress that results in the obvious yield reduction of various crops.

Waterlogging seriously hinders the gas exchange between plant roots and the atmosphere (Striker, 2012). The oxygen in waterlogged soil is rapidly exhausted, resulting in the roots changing from aerobic respiration to anaerobic fermentation, while CO_2_ and ethylene concentrations accumulate. This causes a severe decrease in the ATP synthesis of root cells and impacts multiple metabolic processes of plants (Sairam et al., 2009; Pampana et al., 2016; Kaur et al., 2020). For example, the consequence of damaged root function under waterlogging is stomatal closure, which restricts water and nutrient uptake; this will hinder the influx of carbon dioxide into the leaf as well as transpiration, causing leaf wilting and senescence, in addition to the inhibition of photosynthesis, leading to lower biomass accumulation, thereby reducing kernel weight and grain yield (Ashraf, 2012; Shao et al., 2013; Voesenek et al., 2013; Arguello et al., 2016).

Globally, the responses of crop yield to soil waterlogging have been well documented. For example, several studies have reported that waterlogging events at seedling, jointing, and tillering stages cause different levels of yield reduction in different crop varieties, including cotton (yield decrease range = 23.66–34.79%), wheat (7.75–16.30%), and rice (7.48–57.42%) (Zhang et al., 2012; Zhou et al., 2014; Ding et al., 2020). Moreover, there were also large differences in the change in crop yield in the growth stage under waterlogging (Brisson et al., 2002; De San Celedonio et al., 2014). Wu et al. (2015) concluded that compared with later growth stages, waterlogging treatment at the seedling stage had a greater negative influence on the winter wheat yield. By contrast, Araki et al. (2012) found that waterlogging tolerance was the highest after the anthesis stage, followed by the jointing stage. However, Pampana et al. (2016) demonstrated that no obvious differences in durum wheat yield were observed when waterlogging was applied at the three- and four-leaf stages. In addition to the two abovementioned factors, the experimental duration of waterlogging events also resulted in crop yield reduction; generally, the longer the waterlogging duration, the greater the decrease in crop yield (Ghobadi and Ghobadi, 2010; Zhang et al., 2016; Tian et al., 2020). Clearly, the effect of waterlogging on crop yield differs, which can be attributed to three aspects: crop type, timing, and experimental duration (De San Celedonio et al., 2014; Arduini et al., 2016). However, to our knowledge, there is no comprehensive synthesis on the response of crop yield to the three factors during waterlogging in global regions according to the meta-analysis technique. Therefore, there is an urgent requirement for quantitative and systematic reviews of a global-scale assessment in order to more accurately measure the vital phenological stages and experimental duration when waterlogging events led to the threshold of crop yield reduction. Meta-analysis, a useful tool for quantitative and scientific synthesis, has been used widely to summarize the results from similar studies on a common subject to obtain general conclusions (Werf, 1992; Hedges et al., 1999). Furthermore, it can reveal the specific reasons for the difference between treated and controlled samples (Stanley, 2001; Santachiara et al., 2019). Thus, we used meta-analysis to synthesize the overall effects of waterlogging on crop yield in comparison to well-watered conditions. Likewise, we also provide a profound understanding of the response mechanism of crop yield to waterlogging under different phenological stages, crop types, and durations in the global region. Our specific objectives were to (1) assess the overall change caused by the waterlogging regime on grain yield among different crop types; (2) identify which growth stages have the greatest impact on crop yield during waterlogging; and (3) determine how much waterlogging duration affects crop yield.

## Materials and Methods

### Data Collection

**FIGURE 1:**
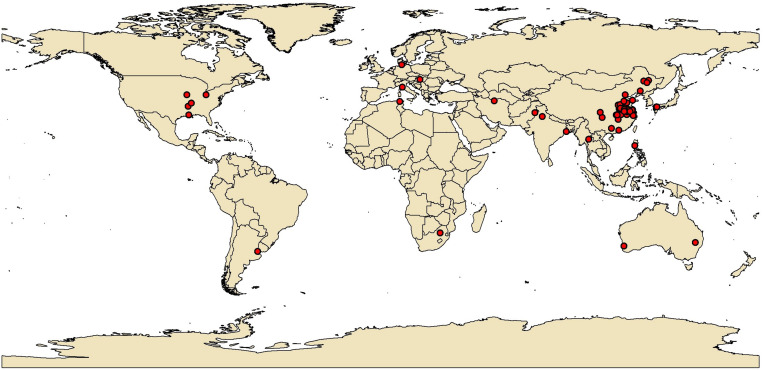
Global distribution of waterlogging experiments used in the meta-analysis.

To investigate the effects of waterlogging in comparison with no waterlogging on the agronomic traits, photosynthetic characteristics, and crop yield, we collected data from peer-reviewed literature and dissertations from 1980 to 2020 based on the Web of Science academic citation database^1^ and the China National Knowledge Infrastructure (CNKI,^2^). The database for this meta-analysis was built based on keywords such as “waterlogging” OR “flooding stress” AND “plant height” OR “biomass” OR “dry weight” OR “net photosynthetic rate (*P*_*n*_)” OR “leaf area index (LAI),” OR “yield.” Only publications that met the following predefined selection criteria were added to the database: (i) studies that involved plants that experienced waterlogging or flooding under field conditions and pot studies; greenhouse experiments were not included; (ii) the experimental research included a waterlogging treatment and a well-watered condition, without other treatments (e.g., growth regulator or the addition of fertilizers); and (iii) the reported plants were field crops; vegetables and horticultural crops were excluded. Based on the above selection criteria, a total of 115 peer-reviewed publications (46 in English and 69 in Chinese) for inclusion with 920 data sets for crop yield, 329 for biomass, 737 for the thousand-grain weight, 242 for plant height, 125 for *P*_*n*_, and 66 for LAI were collected in the global meta-analysis database (Appendix B). These articles originated from 16 different countries (Figure 1 and Supplementary Table A1). The data of crop yield, agronomic traits, and photosynthetic index were derived from tables or were extracted from figures using WebPlotDigitizer (Rohatgi, 2012). Other interrelated information, including location (longitude and latitude), mean annual precipitation (MAP), and basic soil fertility, was also recorded. To clarify the effects of other co-varying factors on crop yield, agronomic traits, and the photosynthetic index under waterlogging regimes, several main categorical variables were categorized into the following groups to facilitate the analysis except the limitation of data availability (Table 1). According to the growth environment of crops, the experimental conditions were categorized as field and pot experiments. Crop types were grouped into five categories: rice, wheat, maize, cotton, and others. Due to the few research articles on other crops under waterlogging stress, mung bean, peanut, soybean, barley, and rape were merged into others. The growth stage of crops was divided into two classes, vegetative stage (VS) and reproductive stage (RS). Waterlogging duration (D, day) was classified into five categories (Amri et al., 2014; Tian et al., 2020): 0 < D ≤ 3, 3 < D ≤ 6, 6 < D ≤ 9, 9 < D ≤ 15, and 15 < D ≤ 28. We categorized water depth (WD, centimeter) into four ranges (Ren et al., 2016; Zhang et al., 2016): 0 < WD ≤ 3, 3 < WD ≤ 5, 5 < WD ≤ 10, and 10 < WD ≤ 20.

**TABLE 1:** Categories used in describing the environmental and management conditions.

**Factors**	**Categories**				
Experimental condition	Field	Pot			
Crop type	Rice	Wheat	Maize	Cotton	Others
Stage	Vegetative stage (VS)		Reproductive stage (RS)		
Duration (D)	0 < D ≤ 3	3 < D ≤ 6	6 < D ≤ 9	9 < D ≤ 15	15 < D ≤ 28
Water depth (cm)	0 < WD ≤ 3	3 < WD ≤ 5	5 < WD ≤ 10	10 < WD ≤ 20	

*D, duration of waterlogging; WD, depth of water layer to be kept during the experiment.*

For crop yield, agronomic traits, and photosynthetic index, the mean (*M*), standard deviations (SD), and sample sizes (*n*) of both waterlogging treatments and the well-watered control were extracted. If only the standard errors (SE) were given, SD was calculated by the following equation: SD = SE × $\sqrt{n}$. When SD and SE were missing, the average coefficient of variation was calculated according to the known mean and standard deviation in all data sets, and then, the missing standard deviation in data sets was calculated (Zhao et al., 2016).

### Data Analysis

To characterize the response of crop yield, agronomic traits, and photosynthetic index to waterlogging, a random-effects meta-analysis was used. We used the natural log of the response ratio (ln *R*) as a measure of effect size (ES):

$$\text{ln}\,R=\text{ln}\,\left(X_{\text{t}}/X_{\text{c}}\right)=\text{ln}\,\left(X_{\text{t}}\right)-\text{ln}\,\left(X_{\text{c}}\right)$$

where *X*_*t*_ and *X*_*c*_ are the measured values of the response variable under the waterlogging treatment and well-watered condition, respectively (Hedges et al., 1999). We used the OpenMEE software (Wallace et al., 2017) to calculate the overall weighted response ratio (ln RR) and to generate bias-corrected 95% confidence intervals (CIs) for the whole data sets and grouped data sets. If the 95% bootstrap CI values did not overlap zero, a significant waterlogging response was considered. To simplify the interpretation, the ES, % was expressed as the percentage change, which was estimated as follows:

Percentage(%)=[exp(lnRR)-1]×100%

Pearson correlation and regression analyses were conducted using Statistical Product and Service Solutions (SPSS v19.0, IBM Corporation, United States) to examine the relationships of the responses of yield versus the various factors. The chi-square test was applied to determine significant differences between groups at *P* < 0.05. To clarify the publication bias, the fail-safe numbers were presented (Supplementary Table A2). A fail-safe number *N* > 5*n* + 10 (*n* is the number of datasets used) indicated the result had no publication bias (Xiang et al., 2017).

## Results

### Overall Response of Agronomic Traits and Photosynthetic Characteristics to Waterlogging

**FIGURE 2:**
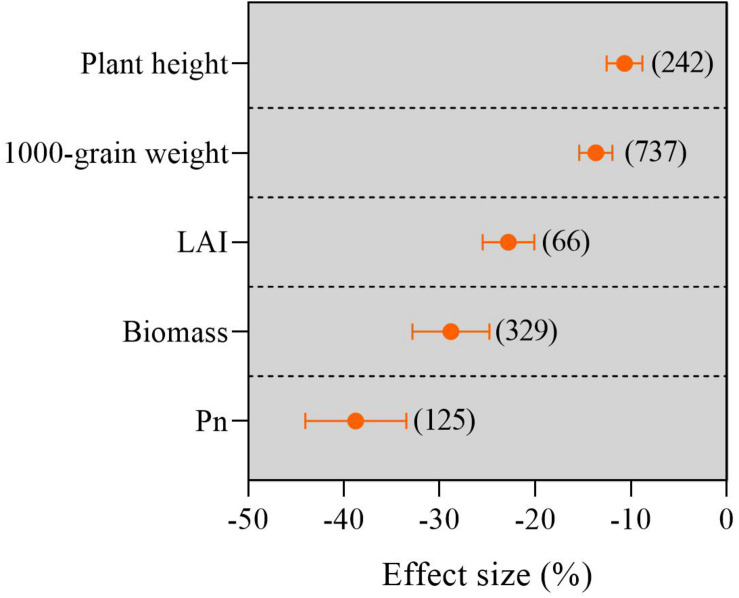
Percentage changes in agronomic traits and photosynthetic characteristics under waterlogging compared to no waterlogging. LAI, leaf area index; *P*_*n*_, photosynthetic rate. The number of observations is displayed in parentheses. The horizontal bar indicates the 95% confidence interval (CI). An error bar that does not overlap 0 indicates a significant increase at *P* < 0.05.

As shown in Figure 2, the waterlogging regime significantly reduced the 1,000-grain weight, plant height, biomass, leaf area index, and net photosynthetic rate (*P*_*n*_). The negative effects of the waterlogging regime on the agronomic traits and photosynthetic characteristics were in the order of *P*_*n*_, biomass, leaf area index, 1,000-grain weight, and plant height. Categorical meta-analysis showed that the waterlogging regime obviously decreased *P*_*n*_, biomass, leaf area index, 1,000-grain weight, and plant height by 39.04, 28.89, 22.89, 13.67, and 10.68%, respectively, compared with no waterlogging.

### Overall Response of Yield Changes Among Different Crop Types Under Waterlogging

**FIGURE 3:**
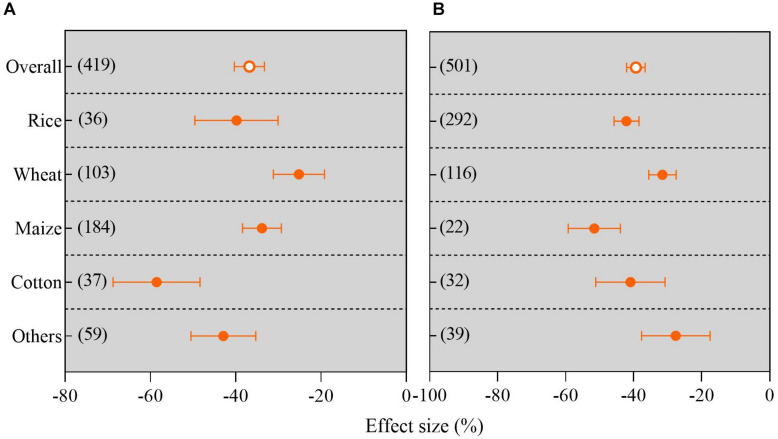
Grain yield response to waterlogging compared with no waterlogging in different crop types: **(A)** field trials; **(B)** pot trials. The number of observations is displayed in parentheses. The horizontal bar indicates the 95% confidence interval (CI). An error bar that does not overlap 0 indicates a significant increase at *P* < 0.05.

The effect of the waterlogging regime on grain yield was significantly affected by the different crop types in the field and pot trials (*P* < 0.05; Figure 3). The results for rice, wheat, maize, cotton, and others did not overlap zero, indicating significant impacts of the waterlogging regime on their yield. Overall, a significantly greater decrease in grain yield was observed in waterlogged pots (39.41%) than under waterlogged field conditions (36.81%) in comparison to no waterlogging. The greatest decrease in cotton yield of 59.95% was observed under waterlogging in comparison to treatments without waterlogging, and wheat had the smallest decrease in grain yield of 25.32% in a field experiment (*P* < 0.05; Figure 3A). The significant decline in grain yield of each crop category under waterlogging followed the order of others (28.04%), wheat (31.61%), cotton (42.07%), rice (42.19%), and maize (51.95%) in the pot experiment (Figure 3B).

### Overall Response of Yield Changes Between Different Growth Stages Under Waterlogging

**FIGURE 4:**
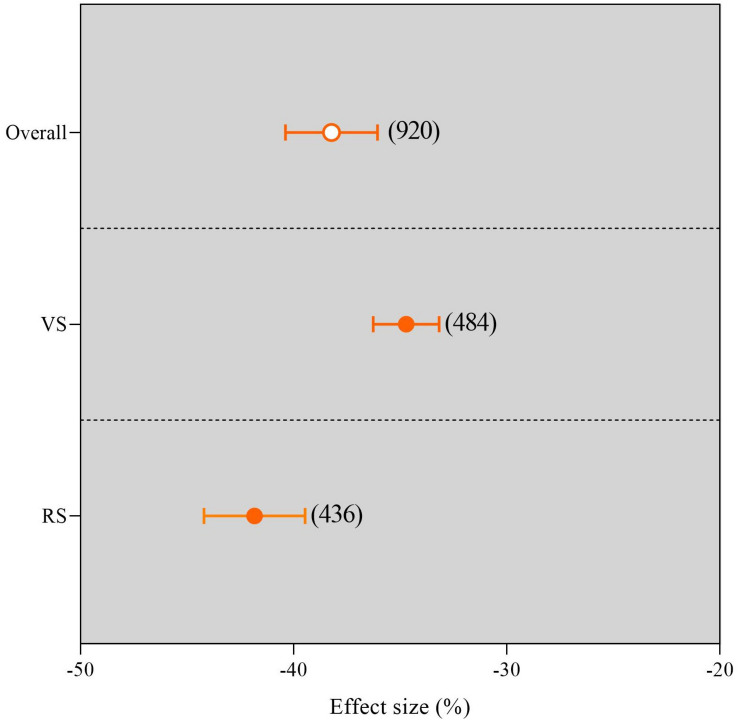
Grain yield response to waterlogging compared with no waterlogging in different growth stages. The number of observations is displayed in parentheses. VS, vegetative stage; RS, reproductive stage. The horizontal bar indicates the 95% confidence interval (CI). An error bar that does not overlap 0 indicates a significant increase at *P* < 0.05.

The grain yield showed clear differences in response to the various phenophase stages (Figure 4). The results show a significant reduction of grain yield in VS and RS under waterlogging conditions (*P* < 0.05). Waterlogging in the VS caused a smaller decline in grain yield than in the RS compared with no waterlogging under field culture (Supplementary Figure 1a). Meanwhile, the yield reduction in the RS under waterlogging was 12.19% higher than that in the vegetative growth stage (Supplementary Figure 1b). Overall, compared with no waterlogging, the reduction in grain yield when waterlogging occurred during the RS (41.90%) was significantly higher than that during the vegetative growth stage (34.75%) (Figure 4).

### Overall Response of Yield Changes With Increasing Experimental Duration Under Waterlogging

**FIGURE 5:**
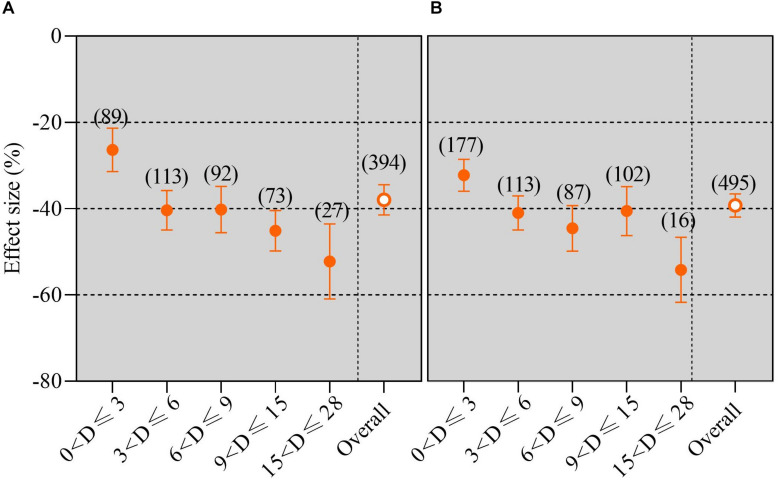
Grain yield response to waterlogging compared with no waterlogging for different experimental durations. The number of observations is displayed in parentheses. The horizontal bar indicates the 95% confidence interval (CI). An error bar that does not overlap 0 indicates a significant increase at *P* < 0.05. **(A)** Field trials; **(B)** pot trials.

**FIGURE 6:**
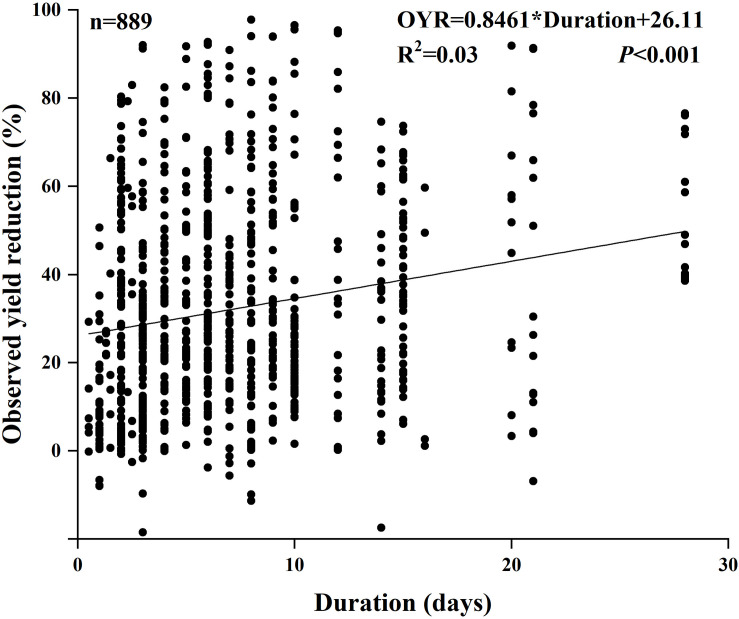
Pearson correlation coefficients between observed yield reduction and experimental duration.

The response of crop yield in all experimental duration subgroups was obviously negative (Figure 5). Furthermore, the crop yield decreased with the extension of the experimental duration. The results of the subgroup meta-analysis indicated that waterlogging duration led to a significant reduction in crop yield ranked as follows: 0–3 days (including day 3), 3–6 days (including day 6), 6–9 days (including day 9), 9–15 days (including day 15), and 15–28 days (including day 28) compared with no waterlogging under field conditions (Figure 5A). The crop yield reduction under the potted waterlogging condition was consistent with that of field waterlogging (Figure 5B). We also found that not only did the extension of waterlogging duration caused a greater reduction of crop yield in the pot trial than in the field trial but also, compared with no waterlogging, the decrease in yield reduction in the RS was greater than that in the VS under waterlogging in pots (Figure 5B, Supplementary Figure 2). The Pearson correlation coefficients showed a significantly positive relationship between the yield reduction and experimental duration (*P* < 0.001; Figure 6).

**FIGURE 7:**
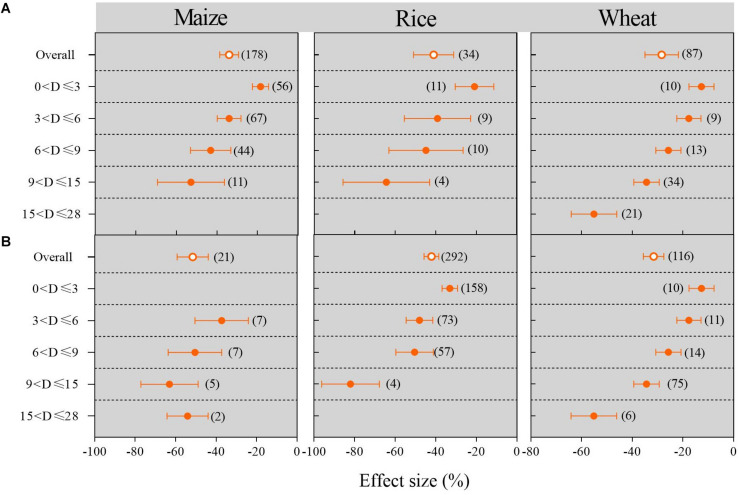
Maize, rice, and wheat yield responses to waterlogging compared with no waterlogging in experiments with different durations: **(A)** field trials; **(B)** pot trials. The number of observations is displayed in parentheses. The horizontal bar indicates the 95% confidence interval (CI). An error bar that does not overlap 0 indicates a significant increase at *P* < 0.05.

The effect of waterlogging duration on maize, rice, and wheat is shown in Figure 7. Irrespective of field or pot conditions, waterlogging always significantly reduced the yield of maize, rice, and wheat, and the longer the waterlogging duration, the lower the crop yield (*P* < 0.05). The meta-analysis results showed that the impact of waterlogging duration on wheat yield was smaller than that on maize and rice compared with no waterlogging. For maize, field waterlogging led to yield decreases of 22.43, 39.77, 52.72, and 76.33 in experiments with durations of 0–3, 3–6, 6–9, and 9–15 days, respectively, in comparison to no waterlogging (Figure 7A). For rice, the yield reduction caused by waterlogging duration was usually similar to that for maize.

## Discussion

### Waterlogging Reduced Crop Yield

This meta-analysis provided a synthetic, quantitative, and systematic analysis of the effects of waterlogging on the 1,000-grain weight, biomass, plant height, LAI, *P*_*n*_, and especially crop yield in the global region based on peer review research. Waterlogging was the main focus of our attention because waterlogging, an abiotic stress, severely limits crop yield in humid areas where continuous rain and poor water infiltration are frequent extreme climate events with global climate change (Yang et al., 2017; Kaur et al., 2020).

In this study, approximately 2.9% of the database samples showed that waterlogging increased crop yield by 7.20% on average in comparison to no waterlogging. This phenomenon may be due to crop varieties that can tolerate longer periods of waterlogging duration or the texture of the experimental soil was relatively loose and the water infiltration was fast (Araki et al., 2012; Wu et al., 2015). However, about 97.1% of the database samples indicated that the yield reduction was less than zero, and its maximum value reached 97.8%. Overall, waterlogging over the global region decreased the crop yield by 32.9% compared with no waterlogging, which is consistent with the previous results reported in other literature (Ploschuk et al., 2018; Ding et al., 2020). This meta-analysis showed that waterlogging caused a decrease in crop yield, and the 1,000-grain weight, biomass, plant height, LAI, and *P*_*n*_ were also reduced. Our Pearson results also indicated that the 1,000-grain weight has a significantly positive correlation with the yield reduction (Supplementary Table A3), which is consistent with previous results (Araki et al., 2012). This can be attributed to the oxygen deficiency induced by waterlogging, which can reduce water absorption by roots, causing a reduction in leaf turgor, LAI, and the net photosynthetic rate, thereby decreasing biomass accumulation and kernel weight and ultimately decreasing crop yield (Wu et al., 2015; Masoni et al., 2016).

### Response of Crop Yield to Waterlogging Varied With the Growth Stage

Numerous studies have confirmed that waterlogging at different phenological stages can decrease crop yield; the magnitude of the decrease in crop yield depends not only on the severity of waterlogging but also on the growth stages. Many studies have explored the vital stage associated with the effect of waterlogging on crop yield and showed that the vegetative growth stage was more tolerant to waterlogging than the RS (Li et al., 2001; Asgari et al., 2012). However, several studies have indicated that the VSs (i.e., seedling stage, stem elongation, and tillering stage) were more susceptible to waterlogging than the RSs (i.e., anthesis and filling stage) (Ren et al., 2017; Wang et al., 2020). In this meta-analysis, we confirmed that waterlogging hazards at different growth stages significantly reduced crop yield, and waterlogging during the RS (41.90%) resulted in a significantly greater yield reduction than that in the vegetative growth stage (34.75%, Figure 4). The reason for this phenomenon may be that waterlogging earlier in the growth stage of the life cycle allows plants to recover through different physiological mechanisms (Setter and Waters, 2003; De San Celedonio et al., 2014). These overall results are in disagreement with our previous conclusion, i.e., the greatest yield reduction in response to waterlogging occurred at the seedling stage, followed by the jointing stage and tasseling stage (Tian et al., 2019).

### Response of Crop Yield to Waterlogging Varied With Crop Type and Experimental Duration

This meta-analysis showed that crop yield after waterlogging was highly dependent on the crop type and experimental duration. The explanation of the effects of waterlogging on crop yield reduction must be prudent and should not ignore the various effects of waterlogging with different durations on different crops. Therefore, an appropriate assessment must be made in terms of the impacts of waterlogging on yield reduction.

In this study, we, for the first time, systematically and comprehensively confirmed that waterlogging significantly reduced the grain yield of rice, maize, wheat, cotton, and other crops. The lowest magnitude of reduction in crop yield was recorded for wheat under field waterlogged conditions. Waterlogging had a smaller impact on wheat under pot conditions. Our results also demonstrated that waterlogging led to the highest reduction in cotton and maize in field and pot trials (Figure 3). Furthermore, we investigated the overall effects of waterlogging duration on crop yield: the meta-analysis results showed that with an increase in the duration of waterlogging, the yields of crops such as maize, rice, and wheat decreased gradually, irrespective of field or pot culture (Figure 7). This is in good agreement with previous studies, e.g., Ren et al. (2016) reported that waterlogging for 6 days caused a greater decline in summer maize yield compared with waterlogging for 3 days, indicating that crop yield is sensitive to the temporal dynamics of experimental duration. We also found that the magnitude of the decrease in wheat yield caused by waterlogging was smaller than that of crops such as maize and rice with an increase in the experimental duration (Figure 7). We speculated that waterlogging induces the formation of more adventitious roots in wheat, which can endure abiotic stress for a longer duration (Deng et al., 2009). Pearson correlation analysis between the water depth under waterlogging and crop yield reduction based on our meta-analysis demonstrated that there was a significant relationship between these variables (*P <* 0.001, Supplementary Figure 3). Similarly, Garssen et al. (2015) also reported that a greater flooding depth negatively affected plant yield.

## Conclusion

The responses of crop yield to waterlogging in a global region were identified based on a meta-analysis. The results indicated that crop yield decreased by an average of 32.9% under waterlogging compared to no waterlogging. The yield loss attributed to waterlogging was influenced significantly by the decrease in the 1,000-grain weight, biomass, plant height, *P*_*n*_, and LAI. Moreover, waterlogging reduced crop yields most significantly in response to crop type, growth stage, and experimental duration. We found a lower sensitivity of wheat yield in response to waterlogging compared to other crops, such as maize, rice, and cotton, irrespective of field or potted conditions. More interestingly, our results also indicated that the reduction in crop yield was impacted by the growth stage subjected to waterlogging. The RS exhibited a greater yield reduction than the vegetative growth stage. Furthermore, the magnitude of the yield reduction caused by waterlogging increased with the experimental duration. In view of the importance of ensuring global crop yields, more attention should be paid to the overall effects of waterlogging on crop yields according to the factors identified in this meta-analysis.

## Author Contributions

B-LF and JL designed the experiment. F-FZ and P-LC contributed to collecting data. FY and YD performed the data analysis and prepared the manuscript. L-XT and Y-CZ wrote the manuscript. All authors contributed to the article and approved the submitted version.

## Conflict of Interest

The authors declare that the research was conducted in the absence of any commercial or financial relationships that could be construed as a potential conflict of interest.

## Footnotes

1 https://apps.webofknowledge.com

2 https://www.cnki.net/
