# Supplementary figures and images for: How Does the Waterlogging Regime Affect Crop Yield? A Global Meta-Analysis
Source: Front Plant Sci. 2021 Feb 19;12:634898. doi: 10.3389/fpls.2021.634898 (PMC7933672; doi:10.3389/fpls.2021.634898)

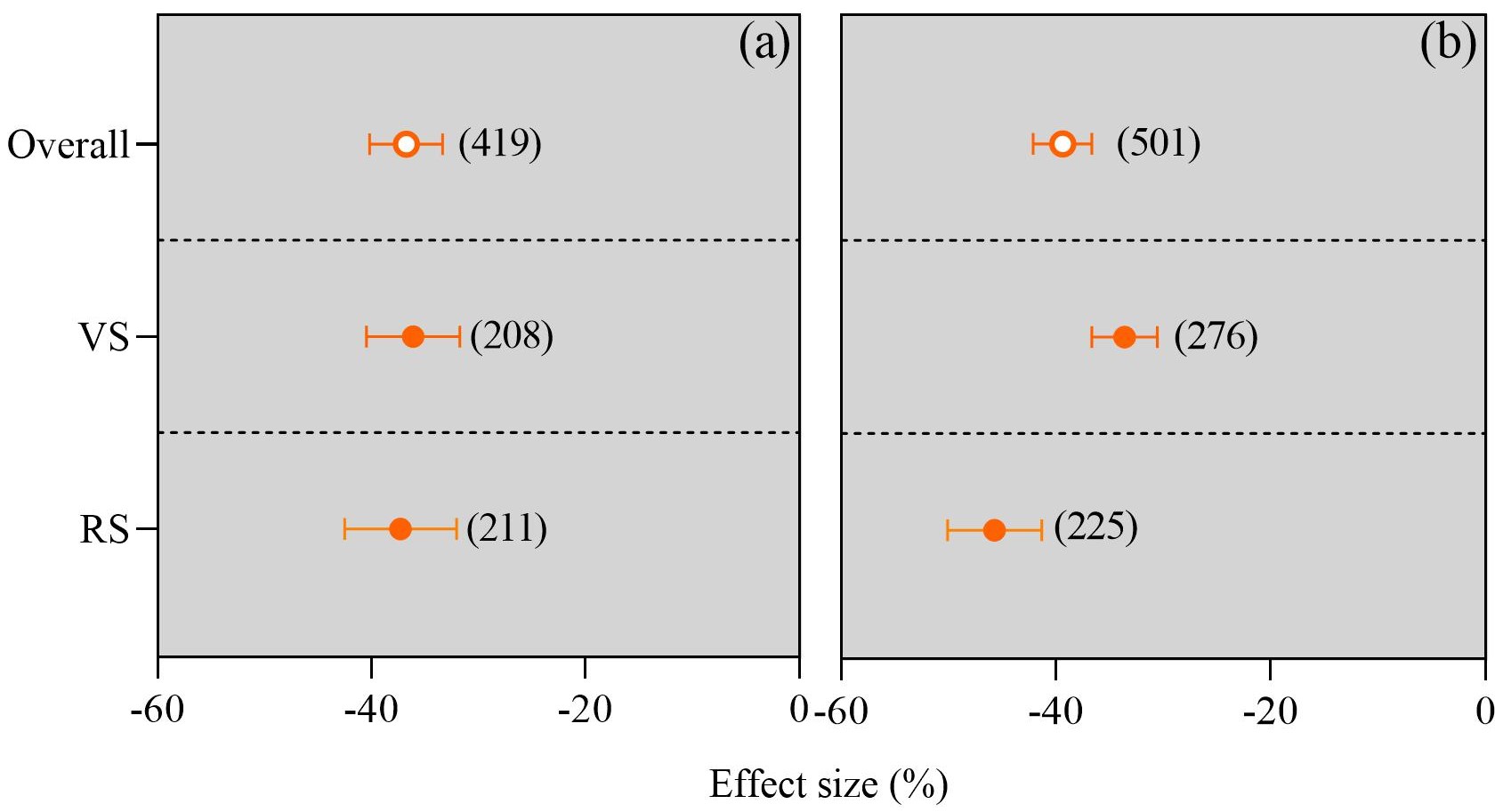

Supplement: Supplementary file 1 [file Image_1.JPEG]

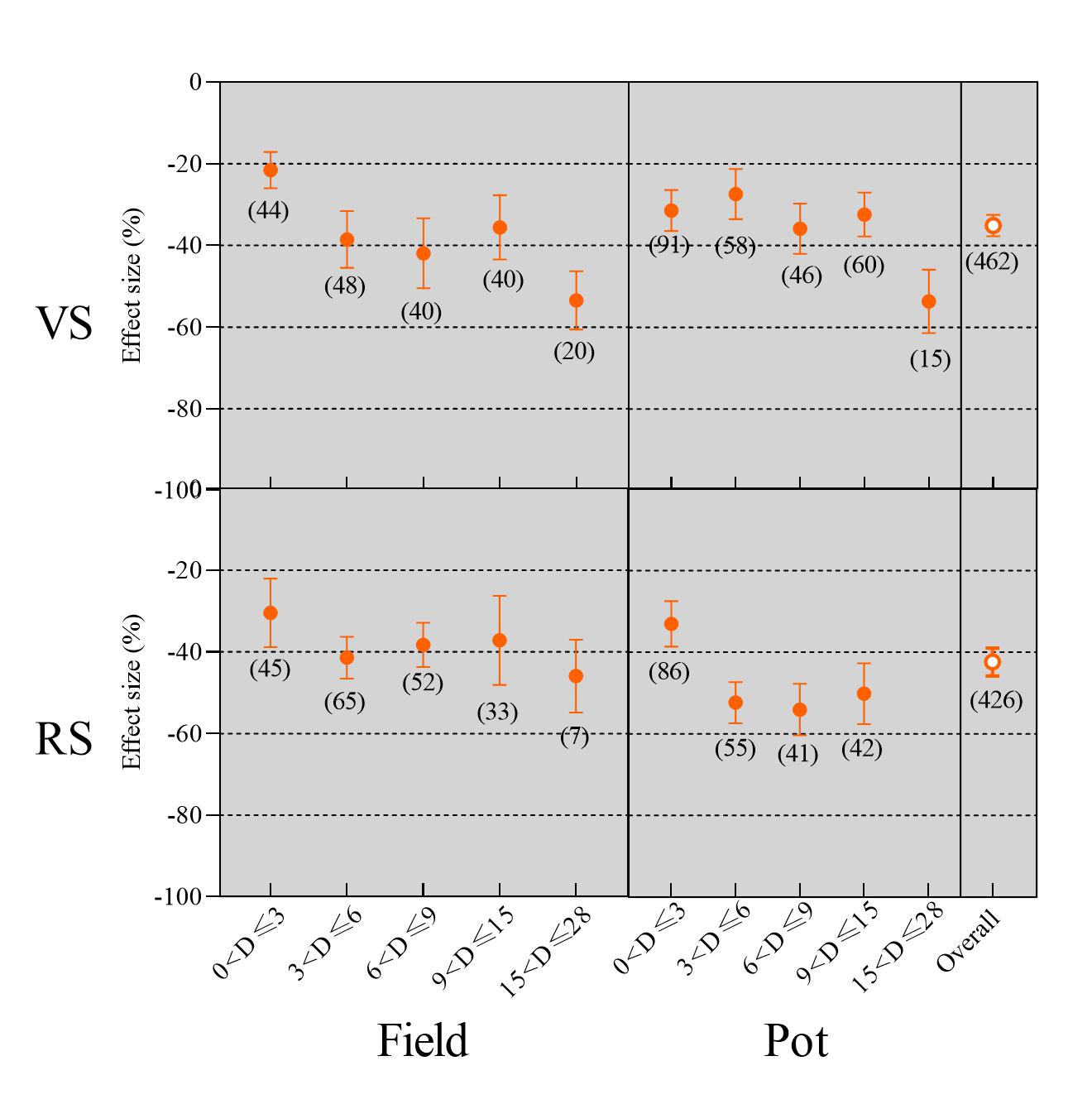

Supplement: Supplementary file 2 [file Image_2.JPEG]

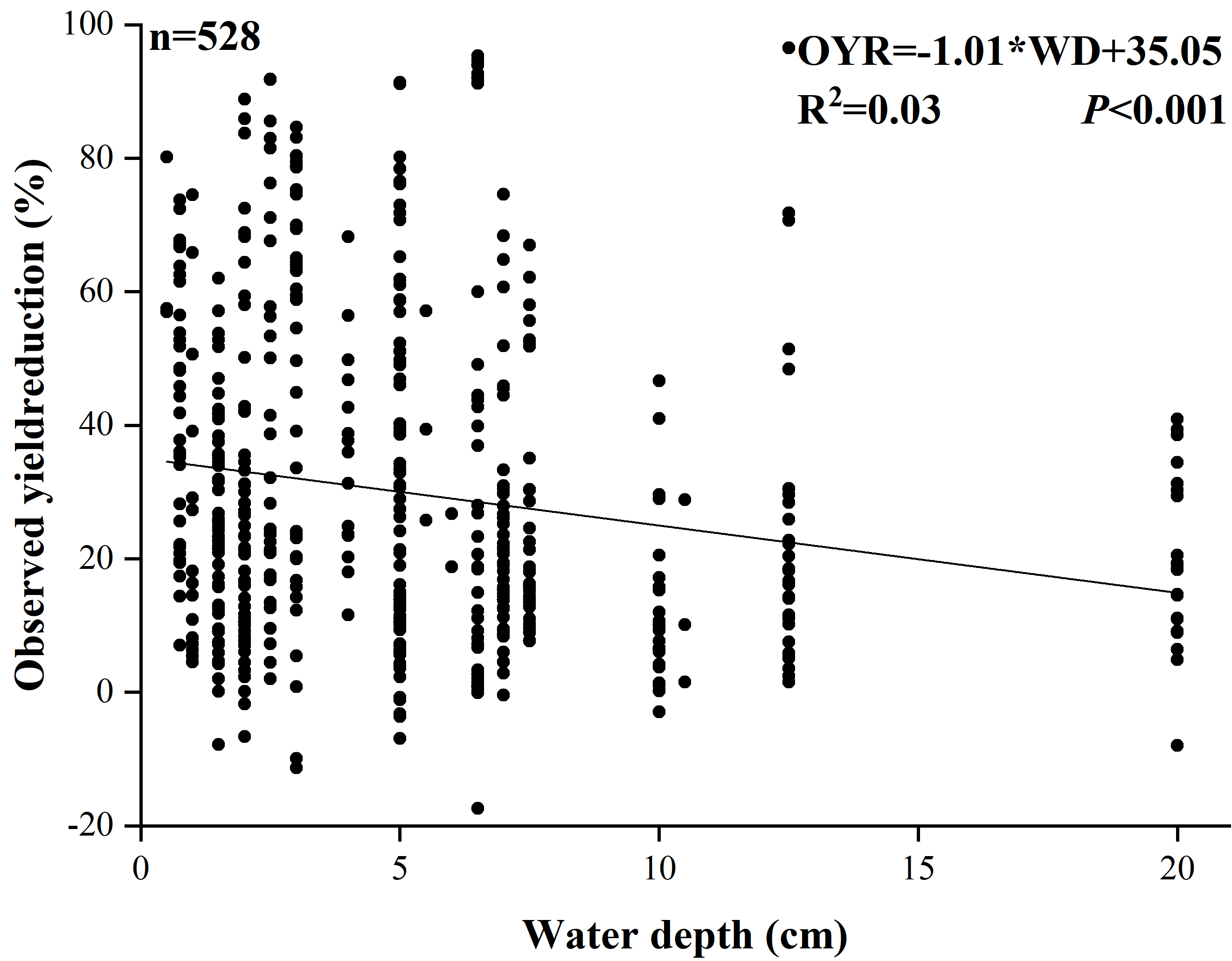

Supplement: Supplementary file 3 [file Image_3.JPEG]
