# Supplementary material for: How Does the Waterlogging Regime Affect Crop Yield? A Global Meta-Analysis
Source: Front Plant Sci. 2021 Feb 19;12:634898. doi: 10.3389/fpls.2021.634898 (PMC7933672; doi:10.3389/fpls.2021.634898)
Supplement: Supplementary file 4 [file Data_Sheet_1.docx]

**Appendix A. Supplementary data**

Table A1 Global distribution of data sets

| Continent | No. of data points | Percentage of total data points |
| --- | --- | --- |
| Asia | 797 | 86.63% |
| Europe | 23 | 2.50% |
| Africa | 24 | 2.61% |
| South America | 12 | 1.30% |
| North America | 52 | 5.65% |
| Oceania | 12 | 1.30% |

**Table A2.** The result of publication bias used in this study (*n* is the number of the cases).

| Index | Yield | 1000-grain weight | Biomass | Plant height | Pn | LAI |
| --- | --- | --- | --- | --- | --- | --- |
| Sample size (*n*) | 920 | 737 | 329 | 242 | 125 | 66 |
| 5*n* + 10 | 4610 | 3695 | 1655 | 1220 | 635 | 340 |
| Fail-safe numbers | 72506742 | 2978775 | 1508416 | 373689 | 657454 | 16143 |
| Observed significance level | < 0.001 | < 0.001 | < 0.001 | < 0.001 | < 0.001 | < 0.001 |
| Target significance level | 0.05 | 0.05 | 0.05 | 0.05 | 0.05 | 0.05 |
| Does bias affect the trend | No | No | No | No | No | No |

Table A3 Pearson analysis of yield and other indicators

| Correlation coefficient | Plant height | Biomass | LAI | 1000-grain weight | Pn |
| --- | --- | --- | --- | --- | --- |
| Yield | 0.341** | 0.507** | 0.145** | 0.500** | 0.562** |
