# Supplementary material for: How Does the Waterlogging Regime Affect Crop Yield? A Global Meta-Analysis
Source: Front Plant Sci. 2021 Feb 19;12:634898. doi: 10.3389/fpls.2021.634898 (PMC7933672; doi:10.3389/fpls.2021.634898)
Supplement: Supplementary file 5 [file Data_Sheet_2.docx]

**Appendix B. The list of 115 publications were extracted for this meta-analysis**

1. Yu Q, Feng, NJ, Wang, SY, et al. (2019) Effects of S3307 on the photosynthesis and yield of mung bean at R1 and R5 stages under waterlogging stress. Acta Agronomica Sinica, 45(7): 1080-1089.(in Chinese)
2. Yu Q. (2019) Effect of uniconazole on waterlogging stress during the reproductive period of mungbean. Daqing, Heilongjiang Bayi Agricultural University. (in Chinese)
3. Ning JH, Lu KD, Huo ZG, et al. (2014) Effects of waterlogging stress on rice morphology and yield component at the jointing stage. Chinese Journal of Ecology, 33(7):1818-1825. (in Chinese)
4. Li CF, Jia CL, Tao ZQ, et al. (2019) Effects of waterlogging at jointing stage on grain yield, plant morphology and dry matter production in different plant height of summer maize. Journal of Maize Sciences, 27(6): 62-67. (in Chinese)
5. Zhen C, Qi DL, Xu Y, et al. (2019) Interactive Effects of nitrogen rate and waterlogging at the jointing stage on growth and yield of spring maize. Journal of Irrigation and Drainage, 38(Supp.1):1-5. (in Chinese)
6. Zhou XG, Han HL, Li CX, et al. (2014) Physiological characters and yield formation of corn (*Zea mays* L.) under waterlogging stress in jointing stage. Transactions of the Chinese Society of Agricultural Engineering, 30(9): 119-125. (in Chinese)
7. Shao CX, Pan XB, Li JW, et al. (2019) Effects of flooding duration in different growth stages on growth and yield component of rice. Transactions of the Chinese Society of Agricultural Engineering, 35(3): 125-133. (in Chinese)
8. Li JC, Dong Q, Yu SL. (2001) Effect of waterlogging at different growth stages on photosynthesis and yield of different wheat cultivars. Acta Agronomica Sinica, 27(4): 434-441. (in Chinese)
9. Ning, JH, Huo ZG, Lu KD, et al. (2013) Effects of water logging on morphological characteristics and yield of hybrid rice during growth stages. Chinese Journal of Agrometeorology, 34(6):678-684. (in Chinese)
10. Zhang F, Wang YY, Zhang JL, et al. (2012) Effects of water-logging at different growing periods on physiological characteristics, pod yield and kernel quality of peanut. Journal of Peanut Science, 41(2):1-7. (in Chinese)
11. Zhou XB, Xiong H, Zhang L, et al. (2013) Effects of different waterlogging stress intensities on the growth of the first mid-season hybrid rice and ratoon rice. China Rice, 19(3): 35-38. (in Chinese)
12. Yu YM, Li, FH, Jiang LX, et al. (2018) Effects of different flooding treatments on growth law and yield of rice in cold region. Journal of Water Resources&Water Engineering, 29(3): 249-253. (in Chinese)
13. Yu, JH. (2016) Effects of different flooding stages and durations on the growth and yield of summer maize. China Rural Water and Hydropower, (3):149-153. (in Chinese)
14. Jiang LN, Xu S, Chang J, et al. (2012) The effect of persistent flooding on the kinetic nutrient absorption and output of wheat. Chinese Agricultural Science Bulletin, 28(27): 113-117.
15. Zhen B, Zhou XG, Lu HF, et al. (2017) How waterlogging occurring at different stages affects the growth and yield of winter wheat. Journal of Irrigation and Drainage, 36(10):46-50. (in Chinese)
16. Liu X. (2011) Effects of different waterlogging stages on emergence rate, yield and quality of winter wheat. Shandong Agricultural University. (in Chinese)
17. Zhao QS, Zhong ZR, Yuan Y. (2017) Effects and countermeasures of waterlogging on growth and yield of rice at regreening stage. China Rice, 23(6):66-68. (in Chinese)
18. Zhang YG, Ning JH, Xie N, et al. (2014) Inﬂuences of waterlogging stress at tillering stage on shape and yield of rice. Hunan agricultural Sciences, (7):14-17. (in Chinese)
19. Li KJ, Shi HF, Shi J, et al. (2007) Effects of waterlogging at tillering stage on growth and yield of rice. Anhui Agricultural Science Bulletin, 13(20): 64-66. (in Chinese)
20. Xuan SL, Shi CL, Zhang JH, et al. (2013) Effects of submergence stress on aboveground matter distribution and yield components of rice at tillering stage. Jiangsu Journal of Agricultural Sciences, 29(6): 1199-1204. (in Chinese)
21. Liu J, Yin XH, Gu AM, et al. (2018) Effects of waterlogging stress on growth traits and yield of rice at tillering stage. Journal of Water Resources and Architectural Engineering, 16(6): 225-229. (in Chinese)
22. Xiang YL, Fang ZS, Zhao JW, et al. (2020) Effect of waterlogging at grain filling stage on grain yield and quality of weak gluten wheat. Journal of Triticeae Crops, 40(6): 730-736. (in Chinese)
23. Wu Q. (2013) Evaluation and analysis of the influence of flood disaster on rice development stage and yield structure. Shanghai Agricultural Science and Technology, (1):30-34. (in Chinese)
24. Yuan HW, Yuan XJ, Tang GM, et al. (2017) Impact of waterlogging at the flower-pod stage on growth and yield of soybean. Journal of Irrigation and Drainage, 36(6):27-30. (in Chinese)
25. Zhang WY, Zhu JQ, Ou GH, et al. (2001) Effects of waterlogging stress at flowering and boll stage on agronomic and economic characters of cotton. China Cotton, 28(9):14-16. (in Chinese)
26. Song XZ, Yang GZ, Luo Z, et al. (2012) Effects of waterlogging at flowering and boll-setting stage on plant growth, some physiological parameters and yield of cotton. China Cotton, 39(9): 5-8. (in Chinese)
27. Yu WD, Feng LP, Sheng SX, et al. (2014) Effect of waterlogging at jointing and tasseling stages on growth and yield of summer maize. Transactions of the Chinese Society of Agricultural Engineering, 30(13): 127-136. (in Chinese)
28. Tian YM, Xu K, Zhou HL, et al. (2013) Effects of post-anthesis waterlogging on flag leaf mesophyll cell and yield in wheat. Meteorological and Environmental Sciences, 36(1): 36-39. (in Chinese)
29. Wu HT. (1999) Effects of waterlogging on growth and yield of early rice. Journal of Zhejiang Agricultural Sciences, (2): 68-71. (in Chinese)
30. Qian L, Wang XG, Luo WB, et al. (2015) Effects of waterlogging stress on morphology and yield of cotton. Transactions of the Chinese Society for Agricultural Machinery, 46(10): 136-143,166. (in Chinese)
31. Zhu. (2014) Effects of waterlogging stress on the growth of loofah seedlings. Hubei Agricultural Sciences, 53(9): 2067-2069. (in Chinese)
32. Wang XS, Deng Z, Zhang WZ, et al. (2017) Effect of timing and duration of flooding on growth and yield of cotton. Journal of Irrigation and Drainage, 36(7): 1-6. (in Chinese)
33. Yu WD, Feng LQ, Hu CD, et al. (2015) Effects of waterlogging during seedling stage on the growth and yield of summer maize in Huang-Huai region. Chinese Journal of Ecology, 34(8): 2161-2166. (in Chinese)
34. Sun QX, Xing HC, Jie YC, et al. (2015) Effect on agronomic traits, yield and straw forage quality of barley by waterlogging simulation in seedling stage. Chinese Agricultural Science Bulletin, 31(15):12-16. (in Chinese)
35. Liu ZG, Liu ZD, Xiao JF, et al. (2013) Waterlogging at seedling and jointing stages inhibits growth and development, reduces yield in summer maize. Transactions of the Chinese Society of Agricultural Engineering, 29(5): 44-52. (in Chinese)
36. Sun XW, Ning JH, Zhang YF, et al. (2015) Impact of submergence stress at milky stage on morphological characteristics and yield of rice. Hunan Agricultural Sciences, (6):27-30. (in Chinese)
37. Wu JG, Liu SZ, Li FR, et al. (1992) Reactions of growth physiology of winter wheat on wet injury. Journal of Henan Agricultural University, 26(1): 31-37. (in Chinese)
38. Wang K, Wang YZ, Tang GM. (2015) Effects of submergence stress on rice yield factors in jointing-booting stage. Journal of Irrigation and Drainage, 34(9): 40-43. (in Chinese)
39. Wang ZX, Li L, Li TT, et al. (2014) Effects of waterflooding on root growth and yield of rice at tillering stage. Journal of Irrigation and Drainage, 33(6):54-57. (in Chinese)
40. Zhang HP, Zhang PP, Li B, et al. (2016) Effects of uniconazole on leaf photosynthetic characteristics and yield of soybean under waterlogging stress. Chinese Journal of Oil Crop Sciences, 38(5): 611-618. (in Chinese)
41. You DL, Zhang X, Yu KK, et al. Effect of exogenous spermidine on growth and physiological properties of maize seedling under waterlogging stress. Journal of Maize Sciences, 24(1): 74-80, 87. (in Chinese)
42. Liu ZD, Xiao JF, Nan JQ, et al. (2010) Effects of waterlogging on morphology, yield and its components of summer maize. Yellow River, 32(12): 157-159. (in Chinese)
43. Yu YM, Li FH, Lian P, et al. (2018) Effects of submergence stress on rice growth in jointing stage. Journal of Water Resources & Water Engineering, 29(6): 240-244. (in Chinese)
44. Liu ZG, Liu ZD, Xiao JF, et al. (2014) Effects of waterlogging stress on photosynthetic characteristic parameters and yield of summer maize. Journal of Irrigation and Drainage, 33(6): 41-46. (in Chinese)
45. Ning JH, Huo ZG, Long ZC, et al. (2013) The preliminary study in rice morphology under waterlogging stress. Chinese Agricultural Science Bulletin, 29(27): 24-29. (in Chinese)
46. Xu DQ, Zheng SF, Wang W, et al. (2016) Effect of waterlogging degree on cotton seedling growth and physiological change. Journal of Agriculture, 6(2): 33-38. (in Chinese)
47. Liu XL, Xu DQ, Zheng SF, et al. (2015) Effects of growth indices of different cotton genotypes and analysis on waterlogging tolerance under field flooding conditions during bud stage and flowering stage. China Cotton, 42(2): 12-16. (in Chinese)
48. Li NL, Guo BJ, Peng KQ, et al. (1998) Effects of flooding treatment on cotton yield and physiological and biochemical characteristics at bud stage. Hunan Agricultural Sciences, (5): 21-22. (in Chinese)
49. Han LL, Zhou Q, Chen WP, et al. (2011) Effects of flooding on growth and yield of soybean. Soybean Sciences, 30(4): 589-595. (in Chinese)
50. Li CX, Zhou XG, Guo DD, et al. Effects of waterflooding on root morphology, photosynthetic characters and yield of winter wheat in grain-filling stage. Journal of Irrigation and Drainage, 32(4): 20-23. (in Chinese)
51. Li XY, Liu ZY, Li TX. (2011) Effects of flood disasters on summer maize’s characters and yield in different areas of henan province. Journal of Anhui Agricultural Sciences, 39(32): 19849-19851. (in Chinese)
52. Ji JH, Huo ZG, Tang LS, et al. (2016) Waterlogging effects on the morphological, physiological characteristics and yield of fresh eating maize. Journal of Maize Sciences, 24(3): 85-91. (in Chinese)
53. Wu QX, Zhu JQ, Yan J, et al. Morphology of middle-season hybrid rice in hubei province and its yield under different waterlogging stresses. Chinese Journal of Agrometeorology, 37(2): 188-198. (in Chinese)
54. Zhou XB, Zhang L, Xiong H, et al. (2014) Effects of submergence stress on growth characteristics and yield formation of mid-season hybrid rice combinations. China Rice, 20(3): 23-29. (in Chinese)
55. Liu ZD, XiaoJF, Feng YH, et al. (2012) Effects of flooding duration and drainage on leaf area and yield of summer maize. Journal of Henan Agricultural Sciences, 41(1): 32-35, 39. (in Chinese)
56. Zhu HX, Jiang LX, Lv JJ, et al. (2019) Effect of waterlogging stress on yield components for rice of frigid region. Journal of Natural Disasters, 28(5): 198-206. (in Chinese)
57. Wang K, Wang YZ, Tang GM. (2010) The effects of waterlogging stress on rape yield factor in flower-pods stage. China Rural Water and Hydropower, (8): 83-85. (in Chinese)
58. Wang K, Xue YF, Wang YZ, et al. (2012) Experimental study of relationship between water and yield of maize. Journal of Irrigation and Drainage, 31(6): 67-70. (in Chinese)
59. Fu QH, Li, JC, Lei M. (2001) Effects of waterlogging stress on nitrogen metabolism and yield in winter wheat at booting stage. Journal of Anhui Agricultural Sciences, 29(5):608-610. (in Chinese)
60. Wu QX, Yang W, Zhu JQ, et al. (2014) Response of hybrid rice to flooding and establishment of drainage index. Resources and Environment in the Yangtze Basin, 23(6): 875-882. (in Chinese)
61. Xu FX, Zhang L, Xiong H, et al. (2016) Relationship between yield loss and flooding during middle and later growth periods in hybrid rice. Acta Agronomica Sinica, 42(9): 1381-1390. (in Chinese)
62. Ji JH, Huo ZG, Tang LS, et al. (2016) Grain yield and quality and physiological and biochemical characteristics of flag leaf in early rice as affected by submergence at filling stage. Chinese Journal of Rice Science, 30(2): 181-192. (in Chinese)
63. Ding DW, Yong BB, Chen JP. (2019) Restoration effect of fertilizers topdressing on growth and yield of flooded maize. Journal of Irrigation and Drainage, 38(12): 37-43. (in Chinese)
64. Guo TZ, Yuan LZ, Zhao YQ, et al. (2014) Effects of waterlogging on maize yield and the rhizosphere soil microorganism. Hubei Agricultural Sciences, 53(3): 505-507. (in Chinese)
65. Hu XG, Yang BB, Shi L, et al. (2018) Effects of waterlogging at jointing stage on photosynthetic characteristics of wheat leaves. Ecological Science, 37(5): 72-76. (in Chinese)
66. Deng LN, Liang T, Zhang ZX, et al. (2015) Effects of waterlogging on photosynthetic characteristics of maize leaves at seedling stage. Journal of Anhui Science and Technology University, 29(6): 41-46. (in Chinese)
67. Xu HT, Wang YH, Xu B, et al. (2108) Impact of water logging on the physiological parameter s and main yield traits of summer maize. Barley and Cereal Sciences, 35(6): 7-11, 29. (in Chinese)
68. Zhou YJ, Wang B, Xu YZ, et al. (2013) Effects of waterlogging stress on growth and yield of early rice at booting stage. China Rice, 19(4): 86-90. (in Chinese)
69. Chen Z, Liang SZ, Wang M, et al. (2017) Effects of simulated flooding stress on yield and leaf characteristics of summer corn. Shandong Agricultural Sciences, 49(4): 26-29. (in Chinese)
70. Amin MR, Karim MA, Islam MR, et al. (2016) Effect of flooding on growth and yield of mungbean genotypes. Bangladesh Journal of Agricultural Research, 41(1): 151-162.
71. Asgari HR, Cirnelis W, Van Damme P. (2012) Wheat (Triticum aestivum L.) growth and yield as influenced by flooding and salinity stresses in Northern Iran. Desert, 17(1): 99-104.
72. Ding JF, Liang P, Wu P, et al. (2020) Identifying the critical stage near anthesis for waterlogging on wheat yield and its components in the Yangtze River Basin, China. Agronomy, 10:130.
73. Ploschuk RA, Miralles DJ, Colmer TD, et al. (2020) Waterlogging differentially affects yield and its components in wheat, barley, rapeseed and field pea depending on the timing of occurrence. Journal of Agronomy and Crop Science, 206(3): 363-375.
74. Ploschuk RA, Miralles DJ, Colmer TD, et al. (2018) Waterlogging of winter crops at early and late stages: impacts on leaf physiology, growth and yield. Frontiers in Plant Science, 9:1863.
75. Tian LX, Bi WS, Ren XS, et al. (2020) Flooding has more adverse effects on the stem structure and yield of spring maize (Zea mays L.) than waterlogging in Northeast China. European Journal of Agronomy, 117:126054.
76. Tian LX, Li J, Bi WS, et al. (2019) Effects of waterlogging stress at different growth stages on the photosynthetic characteristics and grain yield of spring maize (*Zea mays* L.) Under field conditions. Agricultural Water Management, 218: 250–258.
77. Araki H, Hamada A, Hossain MDA, et al. (2012) Waterlogging at jointing and/or after anthesis in wheat induces early leaf senescence and impairs grain filling. Field Crops Research, 137: 27-36.
78. AMri MM, Ouni MHel, and Salem MB. (2014) Waterlogging affect the development, yield and components, chlorophyll content and chlorophyll fluorescence of six bread wheat genotypes (*Triticum aestivum* L.). Bulgarian Journal of Agricultural Science, 20: 647-657.
79. Leul M, Zhou WJ. (1998) Alleviation of waterlogging damage in winter rape by application of uniconazole. Field Crops Research, 59: 121-127.
80. Wang XL, Tang YL, Li CS, et al. (2015) Chlorophyll fluorescence and yield responses of winter wheat to waterlogging at different growth stages. Plant Production Science, 18(3): 284-294.
81. Farkas Z, Varga-László E, Anda A, et al. (2020) Effects of waterlogging, drought and their combination on yield and water-use efficiency of five hungarian winter wheat varieties. Water, 12: 1318.
82. Ren BZ, Zhu YL, Zhang JW, et al. (2016) Effects of spraying exogenous hormone 6-benzyladenine (6-BA) after waterlogging on grain yield and growth of summer maize. Field Crops Research, 188: 96-104.
83. Ding JF, Liang P, Wu P, et al. (2020) Effects of waterlogging on grain yield and associated traits of historic wheat cultivars in the middle and lower reaches of the Yangtze River, China. Field Crops Research, 246: 107695.
84. Zhang, YJ, Chen YZ, Lu HQ, et al. (2016) Growth, lint yield and changes in physiological attributes of cotton under temporal waterlogging. Field Crops Research, 194: 83–93.
85. Gutierrez Boem FH, Lavado RS, Porcelli CA. (1996) Note on the effects of winter and spring waterlogging on growth, chemical composition and yield of rapeseed. Field Crops Research, 47: 175-179.
86. Masoni A, Pampana S and Arduini I. (2016) Barley response to waterlogging duration at tillering. Crop Science, 56: 2722–2730.
87. Wang XY, Liu DM, Wei MM, et al. (2020). Spraying 6-BA could alleviate the harmful impacts of waterlogging on dry matter accumulation and grain yield of wheat. PeerJ, 8:e8193.
88. Wollmer AC, Pitann B, Mühling KH. (2018) Nutrient deficiencies do not contribute to yield loss after waterlogging events in winter wheat (*Triticum aestivum*). Annals of Applied Biology, 173:1-13.
89. Kuai J, Zhou ZG, Wang YH, et al. (2015) The effects of short-term waterlogging on the lint yield and yield components of cotton with respect to boll position. European Journal of Agronomy, 67: 61-74.
90. Alamgir Hossain Md, Araki H, Takahashi T. (2011) Poor grain filling induced by waterlogging is similar to that in abnormal early ripening in wheat in Western Japan. Field Crops Research, 123: 100-108.
91. Saqib M, Akhtar J, Qureshi RH. (2004) Pot study on wheat growth in saline and waterlogged compacted soil. I. Grain yield and yield components. Soil&Tillage Research, 77: 169-177.
92. Kuai K, Liu ZW, Wang YH, et al. (2014) Waterlogging during flowering and boll forming stages affects sucrose metabolism in the leaves subtending the cotton boll and its relationship with boll weight. Plant Science, 223: 79-98.
93. Zhang YJ, Song XZ, Yang GZ, et al. (2015) Physiological and molecular adjustment of cotton to waterlogging at peak-flowering in relation to growth and yield. Field Crops Research 179: 164-172.
94. Islam MR, Hamid A, Khaliq QA, et al. (2010) Effects of soil flooding on roots, photosynthesis and water relations in mungbean (Vigna Radiata (L.) Wilczek). Bangladesh Journal of Botany, 39(2): 241-243.
95. Ren BZ, Zhang JW, Dong ST, et al. (2016) Effects of waterlogging on leaf mesophyll cell ultrastructure and photosynthetic characteristics of summer maize. PLoS ONE, 11(9): e0161424.
96. Liu RX, Yang CQ, Zhang GW, et al. (2015) Root recovery development and activity of cotton plants after waterlogging. Crop ecology & Physiology, 107(6): 2038-2046.
97. Wu JD, Li JC, Wei FZ, et al. (2014) Effects of nitrogen spraying on the post-anthesis stage of winter wheat under waterlogging stress. Acta Physiologiae Plantarum, 36: 207–216.
98. Wu WM, Wang SJ, Chen HJ, et al. (2018) Optimal nitrogen regimes compensate for the impacts of seedlings subjected to waterlogging stress in summer maize. PLoS ONE, 13(10): e0206210.
99. Yordanova RY, Popova LP. (2007) Flooding-induced changes in photosynthesis and oxidative status in maize plants. Acta Physiologiae Plantarum, 29: 535-541.
100. Zheng CF, Jiang D, Liu FL, et al. (2009) Effects of salt and waterlogging stresses and their combination on leaf photosynthesis, chloroplast ATP synthesis, and antioxidant capacity in wheat. Plant Science, 176: 575-582.
101. Zhu M, Li FH, Shi ZS. (2016) Morphological and photosynthetic response of waxy corn inbred line to waterlogging. Photosynthetica, 54(4): 636-640.
102. Tian GL,Qi DL, Zhu JQ, Xu Y. (2020): Effects of nitrogen fertilizer rates and waterlogging on leaf physiological characteristics and grain yield of maize. Archives of Agronomy and Soil Science, 1-13.
103. Yamuangmorn S, Rinsinjoy R, Lordkaew S, et al. (2020) Responses of grain yield and nutrient content to combined zinc and nitrogen fertilizer in upland and wetland rice varieties grown in waterlogged and well-drained condition. Journal of Soil Science and Plant Nutrition, 1-11.
104. Kaur G, Nelson KA, Motavalli PP, et al. (2020) Adaptation to early-season soil waterlogging using different nitrogen fertilizer practices and corn hybrids. Agronomy, 10(3): 378.
105. Arguello MN, Mason RE, Roberts TL, et al. (2016) Performance of soft red winter wheat subjected to field soil waterlogging: Grain yield and yield components. Field Crops Research, 57-64.
106. Bange MP, Milroy SP, Thongbai P. (2004) Growth and yield of cotton in response to waterlogging. Field Crops Research, 88(2-3):129-142.
107. Ferreira JL, Coelho CHM, Paulo César Magalhes, et al. (2007) Genetic variability and morphological modifications in flooding tolerance in maize, variety BRS-4154. Crop Breeding & Applied Biotechnology, 7(3):314-320.
108. Linkemer G, Board JE, Musgrave ME. (1998) Waterlogging effects on growth and yield components in late-planted soybean. Crop Science, 38(6):1576.
109. Kaur G, Zurweller BA, Nelson KA, et al. (2017) Soil waterlogging and n fertilizer management effects on corn and soybean yields. Agronomy Journal, 109(1):97.
110. Najeeb U, Bange MP, Atwell BJ, et al. (2016) Low incident light combined with partial waterlogging impairs photosynthesis and imposes a yield penalty in cotton. Journal of Agronomy and Crop Science, 1-11.
111. Dill TE, Harrison SK, Culman SW, Lindsey AJ. (2020) Grain yield response of corn (*Zea mays* L.) to nitrogen management practices and flooding. Plants, 9, 348.
112. Rhine MD, Stevens G, Shannon G, et al. (2010) Yield and nutritional responses to waterlogging of soybean cultivars. Irrigation Science, 28(2):135-142.
113. Mangani R, Tesfamariam E, Bellocchi G, et al. (2018) Growth, development, leaf gaseous exchange, and grain yield response of maize cultivars to drought and flooding stress. Sustainability, 10, 3492.
114. Yaduvanshi NPS, Setter TL, Sharma SK, et al. (2012) Influence of waterlogging on yield of wheat (*Triticum aestivum*), redox potentials, and concentrations of microelements in different soils in India and Australia. Soil Research, 50, 489-499.
115. Wu JD, Li JC, Wei FZ, et al. (2014) Effects of nitrogen spraying on the post-anthesis stage of winter wheat under waterlogging stress. Acta Physiologiae Plantarum, 36(1):207-216.
